# Prophylactic Intranasal Corticosteroids to Prevent Contact Dermatitis From Ambulatory ECG Monitor Adhesives: A Novel Strategy

**DOI:** 10.7759/cureus.93400

**Published:** 2025-09-28

**Authors:** Noah Y Jo, Loreto L Calaquian, Stephanie A Howes, John-Henry L Dean, Serafim Perdikis

**Affiliations:** 1 Department of Internal Medicine, Malcolm Grow Medical Clinics and Surgery Center, Camp Springs, USA; 2 Division of Cardiology, Brooke Army Medical Center, San Antonio, USA

**Keywords:** ambulatory electrocardiography devices, atrial fibrillation (af), contact allergic dermatitis, fluticasone, remote telemetry

## Abstract

Ambulatory electrocardiogram (AECG) monitors are frequently used to evaluate arrhythmias and assess symptom-rhythm correlation. The extended use of AECG monitors is limited by complications, such as allergic contact dermatitis, secondary to the adhesive. We present the case of a 70-year-old woman with newly diagnosed paroxysmal atrial fibrillation and a known adhesive allergy who developed an allergic contact dermatitis shortly after the application of an AECG monitor. After her symptoms were managed with a topical corticosteroid, fluticasone propionate nasal spray was used as a novel, off-label prophylaxis before the second application of the AECG monitor. The patient then tolerated the full 14-day study without issues, highlighting the potential of aerosolized corticosteroids as a prophylaxis for managing adhesive intolerance in patients requiring long-term AECG monitoring.

## Introduction

Ambulatory electrocardiogram (AECG) monitors are a commonly used non-invasive modality for the evaluation of cardiac symptoms and are used to identify the presence of arrhythmias, quantify disease burden, and establish the presence of symptom-rhythm correlation [1,2]. To fully elucidate the burden of disease, patients are often prescribed these devices for weeks at a time [3-6]. Adhesive intolerance, most commonly in the form of allergic contact dermatitis (ACD), is a significant complication for patients requiring long-term AECG monitoring. Despite attempts to reduce the risk of allergic reactions and improve patient adherence [7], a recent screening study for atrial fibrillation using AECG revealed that 37.9% of users reported limited adverse skin reactions, with 1.2% of patients discontinuing the study due to intolerable side effects [8]. The findings reflect a broader issue inherent to medical adhesives overall [9-11]. While the management strategy of ACD involves allergen avoidance, skin protection, and the use of corticosteroids, there are limited studies that use corticosteroids as a method of prevention for specific medical adhesives. Paret et al. led a study using intranasal fluticasone propionate for the prevention of hypersensitivity to the use of a continuous glucometer [12]; thus, this method was applied for our patient.

We present a case of a patient with a vague history of adhesive allergy that initially prohibited the use of a current-generation AECG monitor. The patient was subsequently pretreated with aerosolized corticosteroid spray at the site of the intended AECG application, which allowed the patient to successfully complete the study.

## Case presentation

Our patient is a 70-year-old woman who presented to the clinic following a recent emergency department visit where she was first diagnosed with atrial fibrillation.

On the day of diagnosis, she was minimally symptomatic, reporting only a vague sense of unease that prompted her evaluation. While on telemetry in the emergency department, she was found to be in atrial fibrillation and spontaneously converted to normal sinus rhythm without intervention. She was discharged with systemic anticoagulation (apixaban 5 mg twice daily), a rate-controlling medication (metoprolol succinate 50 mg daily), and instructions to follow up with her cardiologist.

**Figure 1:**
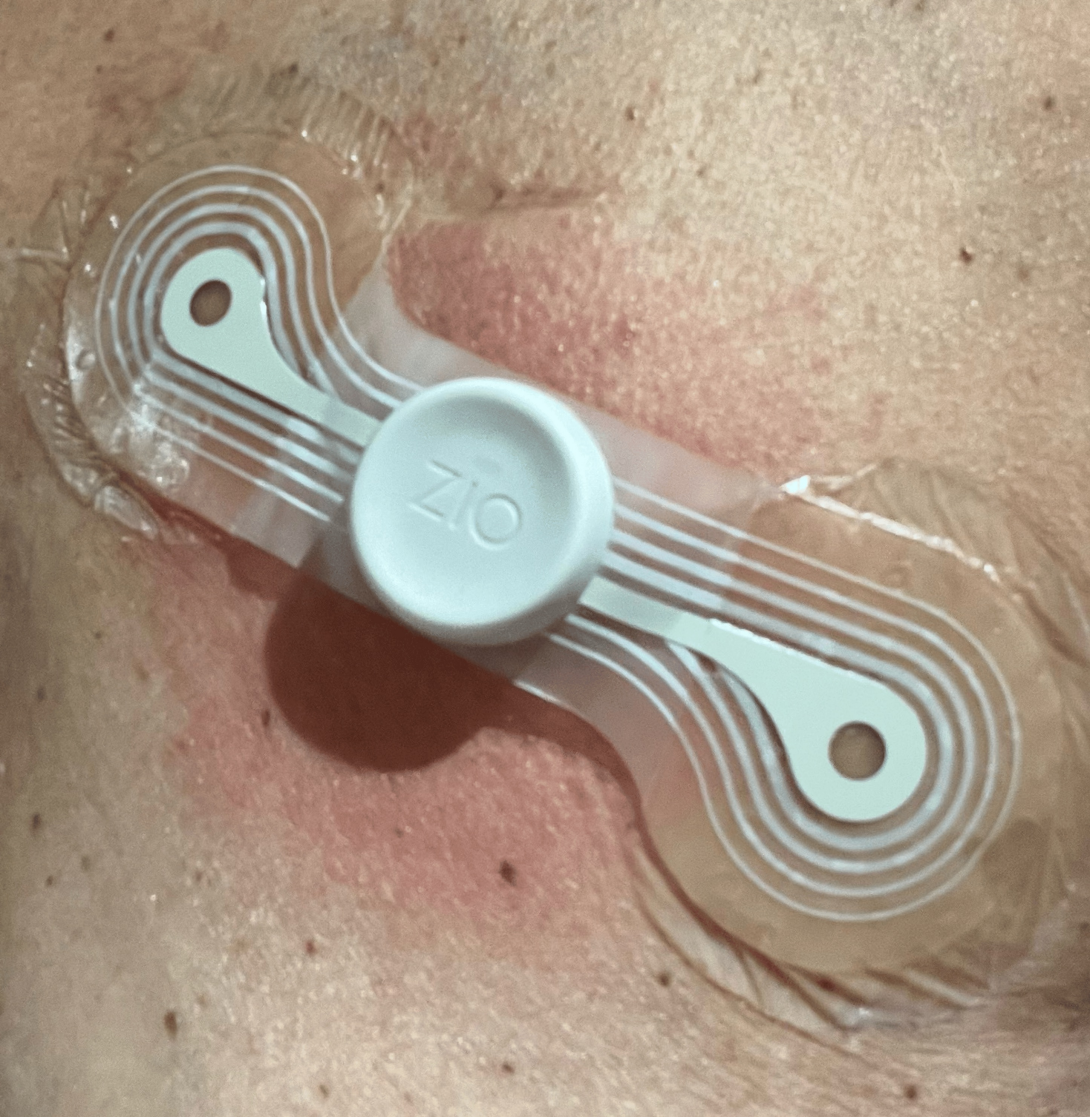
Zio ambulatory electrocardiogram (AECG) monitor overlying affixed to the patient's skin

Following discharge, the patient reported no further symptoms. To better assess her atrial fibrillation burden and identify any subtle symptom-rhythm correlation, she was discharged from the clinic with a 14-day Zio AECG monitor (Figure 1). Within 24 hours, the patient developed intolerable pruritus, erythema, and swelling at the site of AECG monitor placement on the left chest. The device and adhesive were removed, and she was treated with topical hydrocortisone 0.5% cream daily for one week, resulting in complete resolution of her skin reaction. She was not referred to Dermatology for patch testing.

**Figure 2:**
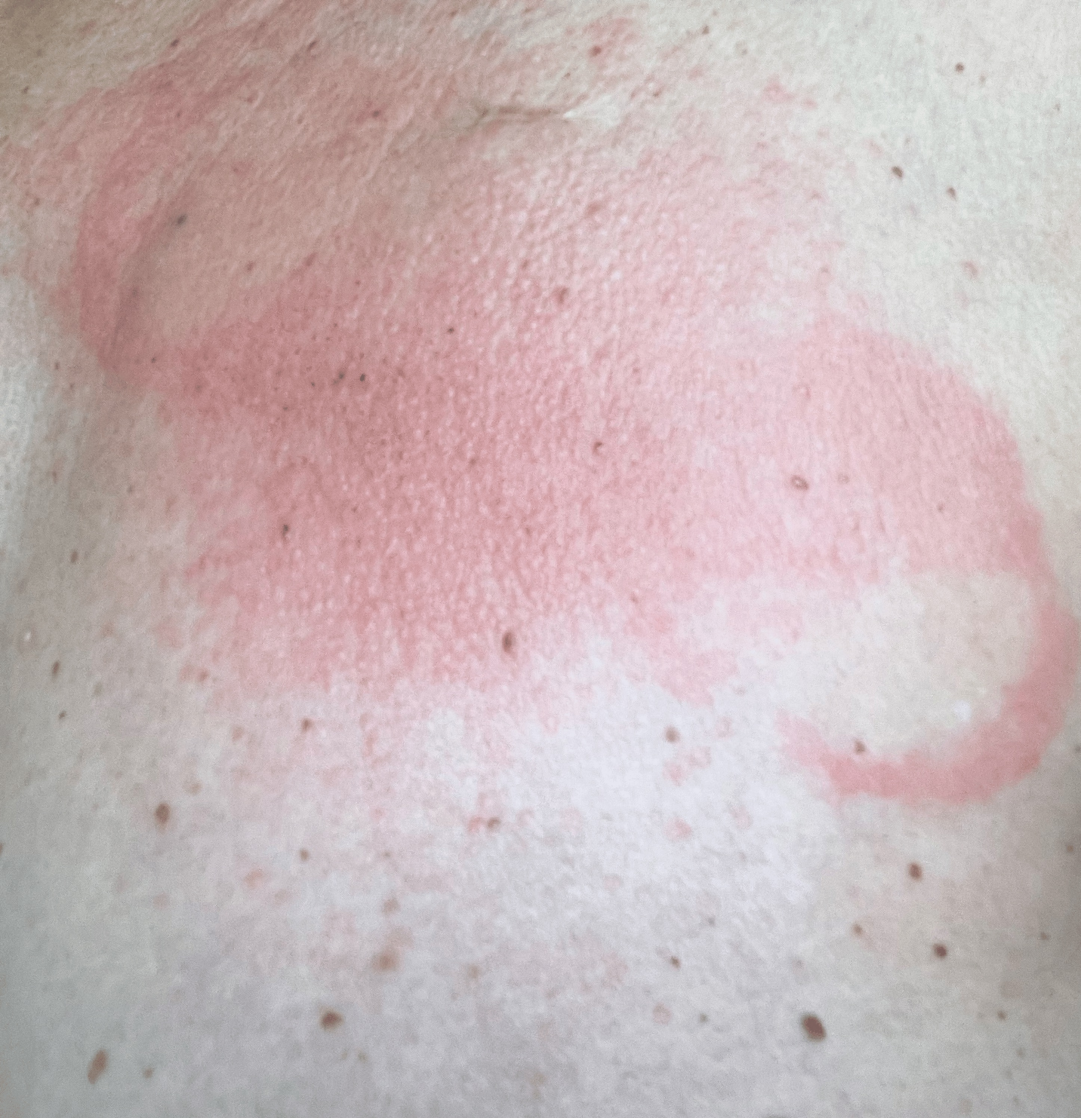
Skin demonstrating allergic contact dermatitis (ACD) following removal of the Zio ambulatory electrocardiogram (AECG) monitor

At her follow-up visit, before reapplication of the AECG monitor, two pumps of intranasal fluticasone propionate 50 mcg spray were applied directly to the uncompromised, fully healed skin of intended AECG placement and allowed to dry. The AECG monitor was then reapplied, and the patient completed a full 14-day monitoring period without recurrence of irritation. The patient's skin was self-monitored during the study (Figure 2).

## Discussion

AECG monitors are useful not only in diagnosing arrhythmias but also in establishing diagnosis, arrhythmia burden, and symptom-rhythm correlation. Guidelines recommend AECG monitoring as a useful diagnostic modality for patients with paroxysmal atrial fibrillation, particularly if episodes or symptoms are infrequent or not captured in the clinic or hospital setting on a 12-lead ECG [1,2]. Extending the wear time of AECG monitors increases the probability of capturing evidence of atrial fibrillation; as such, patch-based AECG monitors are designed to provide continuous telemetry monitoring for up to two weeks [3-6]. Current generation models are designed to reduce the risk of allergic reactions and improve patient adherence [7].

ACD is a type IV hypersensitivity reaction, which is a cell-mediated reaction from the interaction between sensitized T lymphocytes and specific antigens, which leads to an inflammatory reaction from the release of cytokines from CD4+ and CD8+ cells 24 to 72 hours after antigen exposure, ultimately presenting as a delayed-type reaction. The prevalence of ACD has been estimated at around 20% of the general population, according to a systematic review and meta-analysis of studies published between 2007 and 2017 [13]. ACD has been increasingly reported in patients using various types of medical adhesives, including, but not limited to, tapes, dressings, skin adhesives/suture glue, and adhesive devices [9-11]. Although many ingredients, such as acrylates and colophony, have been cited as common allergens in medical adhesive devices causing ACD, identifying the exact culprit antigen may not always be feasible due to a variety of supplemental antigens and materials used in allergens, possible selection bias in patient referral population for patch testing, and user-dependent interpretation of patch testing [10,11,14].

The initial management strategy of ACD involves allergen avoidance and skin protection. Further management is aimed at reducing inflammation. Corticosteroids are commonly utilized as a first-line pharmacologic agent to reduce inflammation. The form (topical vs. systemic) and potency of corticosteroids depend on the location of the lesions and the total body surface area involved [15]. While corticosteroids are predominantly used for the treatment of ACD, some studies have demonstrated reduced reactions to patch tests when pre-treated with corticosteroids, highlighting their potential effectiveness when used as a prophylaxis [16-18]. Additionally, pretreatment of a localized skin area with a topical corticosteroid has shown potential to mitigate the inflammatory response from an antigen [19]. Lastly, the use of aerosolized corticosteroids has also shown potential in preventing dermatitis [20].

This study highlights the potential use of aerosolized corticosteroids as a preventive measure for ACD. The low concentration of corticosteroids in aerosolized products may help reduce the side effects typically associated with traditional topical steroids, including skin thinning, striae, and discoloration. Although this approach has previously been studied for the mitigation of reactions to other medical devices (such as continuous glucose monitors) [12], further research is needed to validate this study on AECG, given that this is a single case. In addition, the lack of a direct comparison of the amount of steroids used between topical and aerosolized forms, standardized skin monitoring, and grading of skin reactions during the study, as well as longitudinal data on the safety profile of the off-label use of intranasal corticosteroids directly on the outer skin, warrants caution prior to broader application.

## Conclusions

This case highlights the increasing challenges of ACD from medical adhesive devices. The successful use of aerosolized corticosteroid in mitigating ACD from AECG underscores the possibility of its use as a prophylaxis. However, further investigation is needed to evaluate the broader applicability of this approach, given that this is a single case of an off-label use of an intranasal corticosteroid lacking longitudinal safety data.
